# Preclinical Potency and Biodistribution Studies of an AAV 5 Vector Expressing Human Interferon-β (ART-I02) for Local Treatment of Patients with Rheumatoid Arthritis

**DOI:** 10.1371/journal.pone.0130612

**Published:** 2015-06-24

**Authors:** Caroline J. Aalbers, Lisette Bevaart, Scott Loiler, Karin de Cortie, J. Fraser Wright, Federico Mingozzi, Paul P. Tak, Margriet J. Vervoordeldonk

**Affiliations:** 1 Arthrogen B.V., Amsterdam, the Netherlands; 2 Division of Clinical Immunology and Rheumatology, Academic Medical Center/University of Amsterdam, Amsterdam, the Netherlands; 3 Center for Cellular and Molecular Therapeutics, The Children's Hospital of Philadelphia, Pennsylvania, United States of America; 4 Department of Pathology and Laboratory Medicine, University of Pennsylvania School of Medicine, Philadelphia, Pennsylvania, United States of America; 5 University Pierre & Marie Curie, Paris, France; 6 Genethon, Evry, France; University of North Carolina at Chapel Hill, UNITED STATES

## Abstract

**Introduction:**

Proof of concept for local gene therapy for the treatment of arthritis with immunomodulatory cytokine interferon beta (IFN-β) has shown promising results in animal models of rheumatoid arthritis (RA). For the treatment of RA patients, we engineered a recombinant adeno-associated serotype 5 vector (rAAV5) encoding human (h)IFN-β under control of a nuclear factor κB promoter (ART-I02).

**Methods:**

The potency of ART-I02 *in vitro* as well as biodistribution *in vivo* in arthritic animals was evaluated to characterize the vector prior to clinical application. ART-I02 expression and bioactivity after transduction was evaluated in fibroblast-like synoviocytes (FLS) from different species. Biodistribution of the vector after local injection was assessed in a rat adjuvant arthritis model through qPCR analysis of vector DNA. In vivo imaging was used to investigate transgene expression and kinetics in a mouse collagen induced arthritis model.

**Results:**

Transduction of RA FLS *in vitro* with ART-I02 resulted in high expression levels of bioactive hIFN-β. Transduction of FLS from rhesus monkeys, rodents and rabbits with ART-I02 showed high transgene expression, and hIFN-β proved bioactive in FLS from rhesus monkeys. Transgene expression and bioactivity in RA FLS were unaltered in the presence of methotrexate. *In vivo*, vector biodistribution analysis in rats after intra-articular injection of ART-I02 demonstrated that the majority of vector DNA remained in the joint (>93%). *In vivo* imaging in mice confirmed local expression of rAAV5 in the knee joint region and demonstrated rapid detectable and sustained expression up until 7 weeks.

**Conclusions:**

These data show that hIFN-β produced by RA FLS transduced with ART-I02 is bioactive and that intra-articular delivery of rAAV5 drives expression of a therapeutic transgene in the joint, with only limited biodistribution of vector DNA to other tissues, supporting progress towards a phase 1 clinical trial for the local treatment of arthritis in patients with RA.

## Introduction

Rheumatoid arthritis (RA) is an immune-mediated inflammatory disease that predominantly affects the joints. Current therapies are aimed at reducing synovial inflammation and pain, and preventing joint destruction by targeting pro-inflammatory cytokines or immune cells like B- and T-cells. Interferon beta (IFN-β) is a cytokine with immunomodulatory properties that also plays a role in bone homeostasis [1]. Of special interest is the ability of IFN-β to reduce production of tumor necrosis factor α (TNF-α), interleukin (IL)-1β, and IL-6, which are all key cytokines in the pathogenesis of RA. Daily systemic treatment with IFN-β protein or a single injection with IFN-β secreting fibroblasts was shown to be beneficial in collagen-induced arthritis (CIA) in mice and rhesus monkeys [2–4]. However, clinical improvement could not be induced using systemic recombinant IFN-β treatment in RA patients when administered 3 times weekly, most likely due to pharmacokinetic issues which resulted in poor bioavailability of the drug [5]. These data suggest that continuous levels of IFN-β at the site of inflammation are required to induce clinical efficacy. Intra-articular gene transfer of IFN-β could provide a solution for this obstacle.

In a proof of principal study in adjuvant-induced arthritis in rats, local delivery of an adenoviral (Ad) vector expressing *rat* IFN-β after disease onset reduced paw swelling, inflammation, and bone and cartilage erosion significantly in both treated and untreated contralateral joints [6]. These results provided a rationale for IFN-β as a therapeutic target for intra-articular gene therapy for arthritis. Recombinant adeno-associated virus (rAAV) was subsequently selected as vector, due to its favorable characteristics, including the ability to induce long-term transgene expression and the efficacy in transducing non-dividing cells [7]. Serotype 5 was selected as the optimal vector for transducing synovial tissue compared to other serotypes [8,9]. In addition, AAV vectors are weakly immunogenic and only a small percentage of the general population as well as RA patients are positive for AAV5 neutralizing antibodies, with the majority of subjects carrying only low titers [10,11].

Using a rAAV5 vector expressing *rat* IFN-β, prolonged therapeutic efficacy was observed in adjuvant-induced arthritis in rats [12]. Therefore, a rAAV5 vector was generated expressing the *human* (h)IFN-β gene under control of a nuclear factor κB (NF-κB) promoter (ART-I02), which is only activated under inflammatory conditions such as during flares of the disease, allowing regulated expression. The aim of this study was to evaluate the potency of ART-I02 *in* vitro as well as biodistribution *in* vivo in arthritic animals to characterize the vector prior to clinical application. Transduction efficacy and potency of ART-I02 was evaluated in a newly developed *in vitro* assay using activated fibroblast-like synoviocytes (FLS), the main target cells of rAAV5 in the joint [8]. In addition the effect of co-medication, methotrexate (MTX), which is used by most of the RA patients included in a phase Ib clinical trial, on transduction efficacy and bioactivity of the therapeutic vector was investigated. Our results show that hIFN-β produced by ART-I02 in FLS from RA patients (RA FLS) is highly bioactive in these cells. Moreover, we demonstrated that FLS from other species, rodents, rabbits and monkeys, can be transduced efficiently but that the bioactivity of human IFN-β is species specific and displays an anti-inflammatory effect only on human and monkey FLS. This was not changed in the presence of MTX.

We then evaluated biodistribution in arthritic and healthy rats after intra-articular injection in the joint. Despite the marked liver tropism [13–15], local injection of ART-I02 in rat showed limited biodistribution to organs other than the joint. In addition, *in vivo* bioluminescence imaging in arthritic mice using a rAAV5 vector expressing luciferase, confirmed local expression of the transgene in the knee joint. Our results support the development of ART-I02 for the local treatment of RA in humans.

## Methods

### Vector Production

ART-I02 was produced as described previously.[16] The plasmid encodes the hIFN-β gene under the control of the NF-κB promoter and a human growth hormone polyadenylation signal. The transgene cassette is flanked by AAV-2 inverted terminal repeats and is packaged in capsid from AAV5 [17]. The vector was purified by combined chromatography and cesium chloride density gradient centrifugation, resulting in empty capsid-free fractions. Vector titers were determined by qPCR using specific primers and probe (Forward primer 5' GCTGGGATTACAGGCGTGAA3', Reverse primer 5' CACGTGGTTACCTACAAAATCAGAA3', MGB Probe 5' 6 FAM ACAGGGAAGGGAGCA BHQ1 3', (Applied Biosystems, Carlsbad, CA, USA)) and expressed as viral genomes/ml (vg/ml). Similarly a rAAV5 vector was produced coding for Firefly Luciferase with a cytomegalovirus (CMV) promoter (rAAV5.CMV.Fluc; Children’s Hospital of Philadelphia, Philadelphia, PA).

### Cell Culture

Human FLS were derived from RA patients through arthroscopy at the rheumatology outpatient department of the Academic Medical Center in Amsterdam, the Netherlands. The study was reviewed and approved by the Academic Medical Center/University of Amsterdam (AMC-UvA) medical research ethics committee (Permit number MEC 07/079). The study was conducted according to the principles outlined in the Guideline for Good Clinical Practice ICH Tripartite Guideline (January 1997). All participants gave written informed consent (according to the Declaration of Helsinki) prior to the study. Tissue was obtained and processed as previously described.[18]

Non human primate (NHP) FLS were derived from joints of one healthy rhesus monkey from a colony at the Biomedical Primate Research Centre (BPRC, Rijswijk, the Netherlands). The cells were obtained from an animal which was sacrificed for the purpose of colony management according to regulations of the Institutional Animal Care and Use Committee of BPRC. BPRC is accredited for the NIH Standard for Human Care and Use of Laboratory Animals. In addition, the BPRC is AAALAC accredited and in full compliance with the EU regulation. The animals are socially housed and offered daily enrichment. The experimental facilities were environmentally controlled and a minimum of 18°C was maintained. These parameters were recorded at least once daily. A 12-hour light/12-hour dark cycle was maintained. The animals were offered a daily diet consisting of monkey food pellets (Hope Farms, Woerden, The Netherlands), bread, fruit and vegetables of the season. Drinking water was provided ad libitum. An overdose of pentobarbital (intravenous injection, 200 mg/kg) after induction of deep sedation/anesthesia with a high dosage of ketamine (intramuscular injection, 10 mg/kg) was used to euthanize the animals, after which synovial tissue was collected from the knee joints.

Mouse FLS were derived from mice with CIA. The mouse study was reviewed and approved by the animal care and use committee of the University of Amsterdam (Amsterdam, The Netherlands; Permit number DRI 102086). Furthermore, an immortalized cell line (HIG-82, ATTC, Middlesex, UK) of rabbit origin and a rat dermal fibroblast cell line (RDF, Cell Applications, San Diego, CA, USA) were used. RA, mouse and NHP FLS were cultured as monolayers in Dulbecco’s modified Eagle medium (DMEM), supplemented with 10% fetal calf serum (FCS), penicillin-streptomycin, L-glutamine, HEPES and gentamycin and maintained in a 37°C incubator at 5% CO2. RDF cells were cultured in similar medium, supplemented with 40% FCS. HIG-82 cells were cultured in Ham’s F12 medium, supplemented with 10% FCS and penicillin-streptomycin.

### In Vitro Transduction Experiments and Transgene Expression Analysis

Twenty-four hours prior to transduction cells were seeded in 48-well plates at 15,000 cells per well. Infections were performed at a vector dose of 200,000 vg/cell in DMEM/Ham’s F12 medium. The proteasome inhibitor doxorubicin (DOX; 0.4 μM) was added to culture media, first upon seeding compared to addition four hours post-transduction to test optimal conditions, in further experiments four hours post-transduction only. To activate the NF-κB promoter, cells were stimulated with species-specific TNF-α (1 ng/ml) with or without IL-1β (10 ng/ml) 24 hours post-transduction. For NHP FLS human TNF-α was used. Supernatants were harvested 48 hours later and stored at <−70°C until further analysis. Conditions were tested in triplicate, experiments were repeated 2 to 6 times. The effect of methotrexate (MTX) on transduction, transgene expression and bioactivity was analyzed by adding MTX (emthexate PF, Pharmachemie, Haarlem, Netherlands) in 3 concentrations (10 nM, 1 μM, 100 μM) upon seeding of cells, in combination with DOX addition 4 hours post-transduction and with stimulation 24 hours post-transduction.

Levels of human IL-1 receptor antagonist (ra), IL-6, IL-8, MMP-3, mouse IL-6, rat IL-6 and rabbit IL-8 were determined using sandwich ELISAs (Duoset, R&D systems, Minneapolis, MN, USA). Monkey IL-6 and IL-8 concentrations were determined using commercially available sandwich ELISAs (U-CyTech Biosciences, Utrecht, Netherlands) and hIFN-β levels were detected by a Verikine hIFN-β ELISA kit from PBL Interferon Source (Piscataway, NJ, USA, cat. #41410). All were performed according to manufacturer’s protocols. Interferon β bioactivity was determined using a quantitative gene reporter bioassay (iLite alphabeta kit, Biomonitor, Copenhagen, Denmark). Human type 1 IFN-sensitive cells from the bioassay were stimulated with supernatants generated by transduction of RA-FLS and NHP-FLS as described above. Interferon bioactivity was measured by degree of luminescence and expressed as arbitrary units. The bioassay was used according to manufacturer’s protocol. Briefly, all kit components were thawed at room temperature, except for the human type 1 IFN-sensitive cells, which were rapidly thawed at 37°C prior to use. Samples and standard curve were prepared in desired dilutions. Cells, diluent, standard curve, samples and control samples were added to a white-walled micro well plate, which was incubated at 37°C, 5% CO2 in the dark for 7 hours. After addition of substrate in assay buffer luminescence was determined at a luminometer (Synergy HT multi-mode microplate reader, Biotek, Winooski, VT, USA). In addition to the protocol, a hIFN-β standard curve (hIFN-β ELISA kit) was used to obtain a more accurate standard curve for hIFN-β.

An overview of all transduction experiments performed is shown in S1 Table.

### In Vivo Biodistribution Experiment in Rats

To study the biodistribution of the ART-I02 vector after local injection, arthritic and non-arthritic rats were injected in the right ankle joint and sacrificed one and four weeks later (S2 Table). The study was reviewed and approved by the animal care and use committee of the University of Amsterdam

(Amsterdam, The Netherlands; Permit Number: DSK 1000, DRI 101280). The study was carried out in strict accordance with the recommendations in the Dutch Law on Animal Welfare (Wet op Dierproeven) and all efforts were made to minimize suffering. Animals were maintained under pathogen-free conditions in the animal facility of the University of Amsterdam. Adjuvant arthritis was induced in male Lewis rats (150–200 gram; Harlan Sprague Dawley, Horst, The Netherlands) in 4 groups (n = 6 per group) by intradermal injection at the base of the tail with 1 mg Mycobacterium tuberculosis (Brunschwig Chemie, Amsterdam, Netherlands, cat #H37RA) in 0.1 ml mineral oil.[19] Two groups were not immunized. Vectors were injected in all groups on day 14 after immunization. Both procedures were performed under isoflurane anaesthesia. Four groups received ART-I02 in the right ankle (total of 6x10^11^ vg in 20 μl of 3x10^13^ vg/ml), one group received ART-I02 intravenously (6x10^11^ vg in 200 μl). As control vector, one group received rAAV5-CMV-GFP in the right ankle (8x10^10^ vg in 20 μl of 4x10^12^ vg/ml). Two groups with ART-I02 (with and without arthritis) were sacrificed one week after vector administration, the other groups four weeks after vector administration by carbon dioxide inhalation. Strict tissue collection cleaning procedures were followed and blood, hind paws and organs were collected in such order that organs least likely to contain AAV5 genomes were collected first in order to minimize the potential for crosscontamination (distal from the joints). Blood was processed to plasma. Ankle joints were collected, with the feet just below the ankle joint and the leg above the ankle joint removed. Thereafter, skin was removed, and the remaining tissue (ankle joint including surrounding peripheral tissue) was snap frozen in liquid nitrogen, pulverized using a pestle and mortar, and homogenized in Trizol Reagent (100 mg/ml; Invitrogen, Carlsbad, CA, USA) using a tissue homogenizer. DNA from blood and tissues was isolated using a DNeasy Blood and Tissue Kit (#69506, Qiagen, Hilden, Germany) according to kit protocol. A sensitive real-time polymerase chain reaction (RT-PCR) was used to detect viral DNA sequences in organs and blood. Briefly, RT-PCR amplification was performed in a total volume of 10 ul containing 100 ng sample DNA, 2x Thermo mix (5 μl, Thermo, Waltham, MA, USA), hIFN probe (5’CCTCCAAATTGCTCTCCTGTTGTGC3’, 20 pmol/μl, Taqman probe, Biolegio, Nijmegen, Netherlands) and hIFN *rev* (5’GGAATCCAAGCAAGTTGTAGC3’, 20 pmol/μl, Bolegio) and hIFN *for* (5’CGGATCTCTAGAATGACCAACA3’, 20 pmol/μl, Bolegio) primers. GAPDH was used as an internal reference gene. Reactions were run on a robot-PCR-system (Roche, Basel, Switzerland). The thermal profile consisted of 1 cycle at 50°C for 2 minutes, followed by 1 cycle at 95°C for 10 min and 40 cycles at 95°C for 15 seconds and 60°C for 1 minute. The assay performed included (in triplicate) a standard curve of seven serial dilutions of rAAV5-NFkB-hIFNb and a no-template control. The lower limit of sensitivity for this assay was determined to be 100 viral copies/ 100 ng gDNA.

### In Vivo Imaging Experiments in Mice

To study the transgene expression and kinetics of a rAAV5 vector after local injection, arthritic mice were injected with rAAV5.CMV.Fluc in both knee joints and sacrificed seven weeks later (S3 Table). Collagen induced arthritis was induced in male DBA mice (8–12 week old; Harlan Sprague Dawley, Horst, The Netherlands) (n = 5) by means of an intradermal injection of 100 μl collagen type II (2 mg/ml), diluted 1:1 in CFA (mineral oil and heat-killed M. Tuberculosis 2 mg/ml) (Chondrex Inc., Redmond, WA, USA). On day 21 a booster injection was administered intraperitoneally containing 100 μg collagen type II dissolved in 100 μl NaCl. The vector was injected on day 21 after immunization in both knees, at the onset of disease. Animals received 5 μl of 3.42x10^13^ vg/ml per knee joint (3.42x10^11^ vg total). Immunization and vector injections were performed under isoflurane anaesthesia.

Luciferase expression was measured 3 days and then weekly until 7 weeks after vector administration. Mice were anaesthetized with 3% isoflurane and oxygen. D-luciferin potassium-salt substrate (Caliper Life Sciences, Hopkinton, MA, USA) was injected intraperitoneally (150 mg/kg of body weight, in a volume of approximately 200 μl). Photon counts were acquired 10 minutes after substrate administration for 5 minutes using a cooled charge-coupled device (CCD) camera system (Photon Imager, Biospace Lab, Paris, France). Light surface images were obtained immediately after each photon counting session to provide an anatomical view of the animal. Image processing and signal intensity quantification and analysis were performed using M3 Vision (Biospace Lab). Images were displayed as a pseudo-color photon count image, superimposed on a gray scale anatomic white-light image, allowing assessment of both bioluminescence intensity and its anatomical source. Regions of interest (ROI) were defined by drawing an elliptical ROI over the knee joint region. The surface area of the ROI was kept constant. The number of photons emitted per second per square centimetre per steradian was calculated as a measure of luciferase activity. Animals were sacrificed seven weeks after vector administration by cardiac puncture under isoflurane anaesthesia, followed by cervical dislocation. The study was reviewed and approved by the animal care and use committee of the University of Amsterdam (Amsterdam, The Netherlands; Permit Number: ART 102659) and carried out in strict accordance with the recommendations in the Dutch Law on Animal Welfare (Wet op Dierproeven). Animals were maintained under pathogen-free conditions in the animal facility of the University of Amsterdam.

### Statistical Analysis

Statistical evaluation for *in vitro* results was performed using factor correction to correct for between-session variation followed by nested ANOVA.[20] RT-PCR data were analysed using robot-PCR software (Roche) and Graphpad Prism (La Jolla, CA, USA). For all tests, differences with a p-value of <0.05 were considered significant.

## Results and Discussion

### Vector Potency In Vitro in Fibroblast-Like Synoviocytes

#### In vitro FLS transduction assay

**Fig 1:**
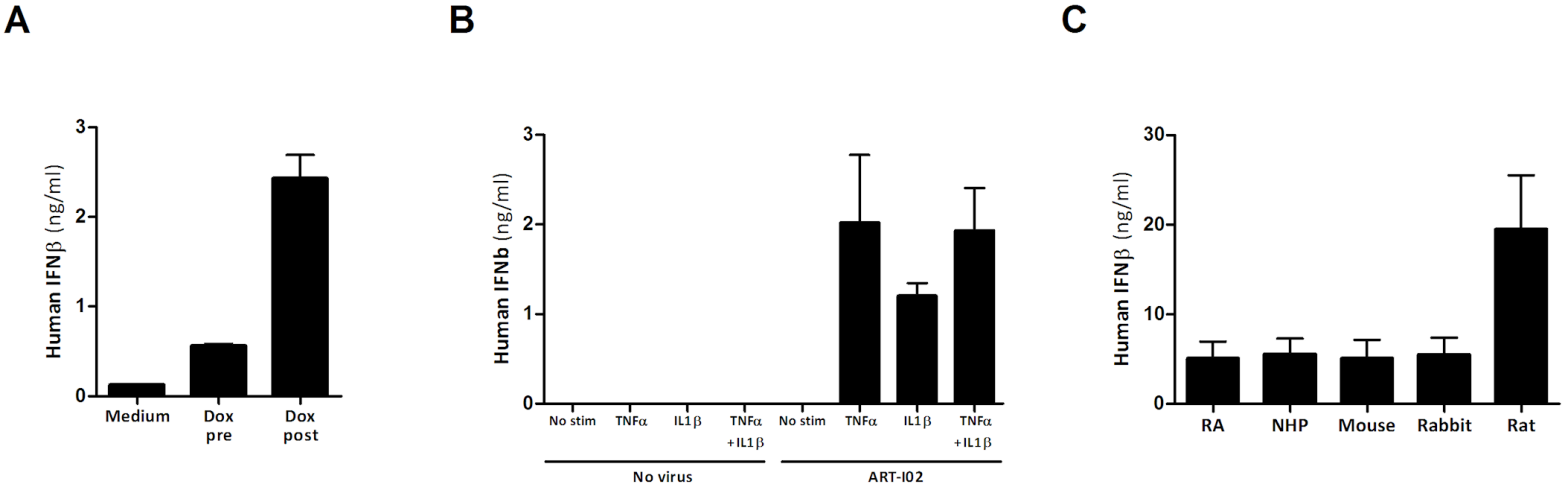
Human IFN-β expression levels in RA, rodent, rabbit and NHP FLS. Comparison of hIFN-β expression levels showed that DOX addition 4 hours post-transduction (‘Dox post’) improved expression in RA FLS more than 4-fold compared to DOX treatment 24 hours pre-transduction (‘Dox pre’) and almost 20-fold compared to medium only. In all three conditions, cells were stimulated with TNF-α (A). Transduction with ART-I02 resulted in increased levels of hIFN-β after stimulation of RA FLS. Without addition of ART-I02 or without stimulation (TNF-α and/or IL1-β), no hIFN-β was detected (B). Double stimulation with TNF-α and IL-1β did not increase the level of transgene expression compared to TNF-α stimulation alone (B). Human IFN-β production was detectable in culture supernatants of FLS of all species (C). For panel B and C, in all conditions doxorubicin was used. Data shown are mean + SEM, N = 2–3 experiments.

*In vitro*, AAV has demonstrated low transduction efficiency [21], with one limiting step being intracellular processing. Doxorubicin, an anthracycline derivative, has been shown to increase transduction efficiency for several AAV serotypes in different cell lines, through a proteasome modulating mechanism [22–24]. However, whether DOX is able to enhance rAAV5 transduction in primary slowly-dividing RA FLS has never been tested. Recombinant AAV5 transduction in response to two different transduction schemes (24 hours pre-transduction compared to 4 hours post-transduction) was evaluated in RA FLS. Addition of DOX 4 hours post-transduction improved ART-I02 transduction in RA FLS 4-fold compared to DOX treatment 24 hours pre-transduction, independent of the presence TNF-α or TNF-α and IL-1β stimulation (Fig 1A). Further experiments were conducted with the addition of DOX 4 hours post-transduction.

#### Vector expression and bioactivity

**Fig 2:**
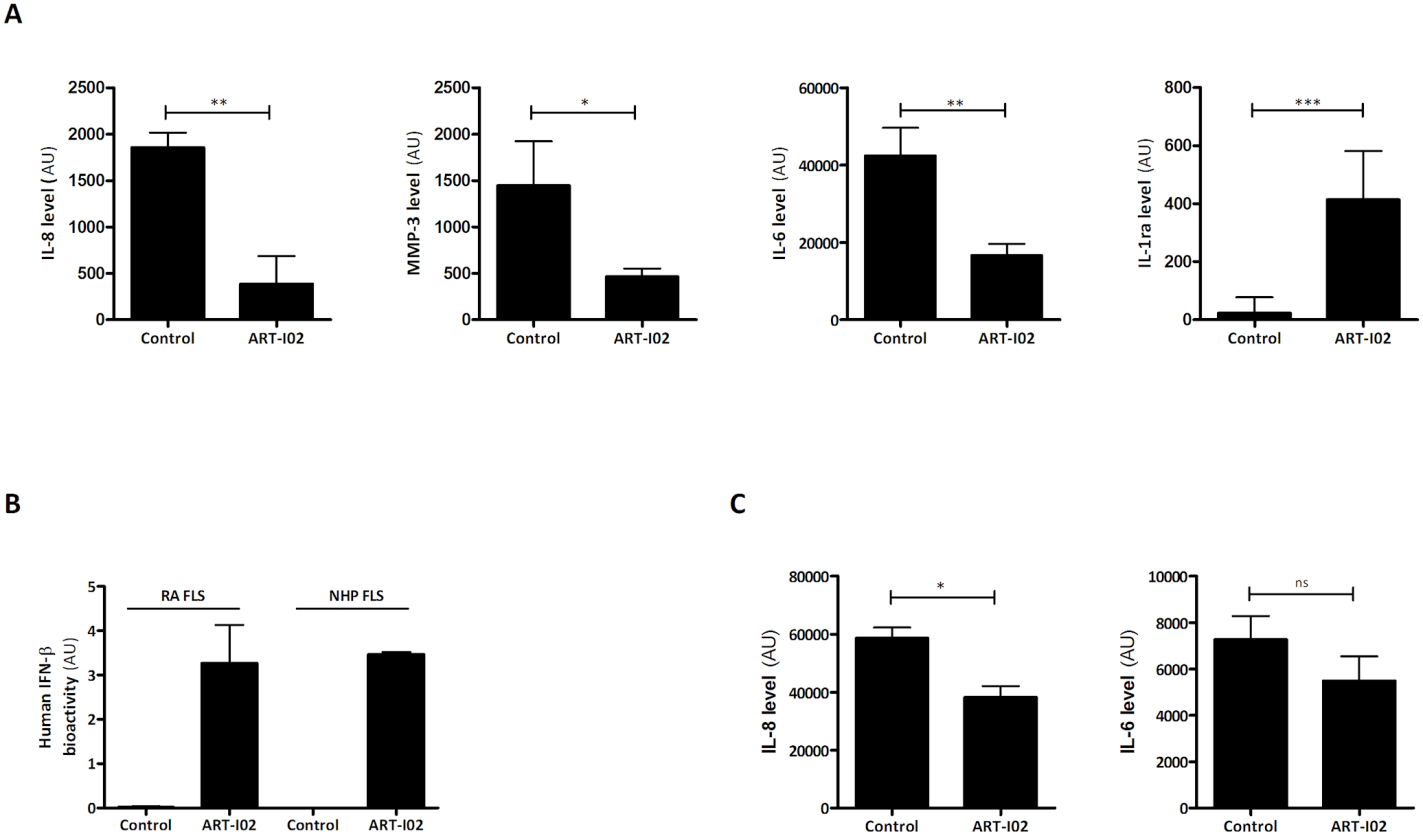
Bioactivity of ART-I02 in human and NHP FLS. In the presence of hIFN-β the anti-inflammatory cytokine IL-1ra was up regulated in RA FLS (A). Levels of pro-inflammatory cytokines (IL-6, IL-8) as well as MMP-3 were downregulated in RA FLS (A). A quantitative gene reporter bioassay confirmed the presence of bioactive hIFN-β after transduction of ART-I02 in combination with stimulation both in RA FLS as well as NHP FLS (B). In NHP FLS (C), IL-8 was also downregulated significantly, although IL-6 in NHP FLS showed only a trend towards downregulation. All samples were stimulated with TNF-α, except for the human IL-6 data, where samples were stimulated with TNF-α and IL-1β. Control cells were stimulated but not transduced with ART-I02. Data shown are mean + SD, N = 2–6 experiments, * indicates P<0.05, ** P<0.01 and *** P<0.001.

The capability of ART-I02 to transduce FLS from different species and induce hIFN-β production was assessed *in vitro*, in order to investigate rAAV5 tropism for FLS. Transduction of RA FLS with ART-I02 resulted in increased levels of hIFN-β after stimulation (Fig 1B). Stimulation with TNF-α and IL-1β did not increase hIFN-β levels compared to TNF-α alone, and transgene expression was not detected in the absence of stimulation or the absence of ART-I02 (Fig 1B). ART-102 gene expression following transduction with ART-I02 was investigated in rat, murine, rabbit and NHP FLS. Human IFN-β production was detectable in supernatants of FLS of all species, with hIFN-β levels comparable to levels produced by RA FLS for NHP, rabbit and mouse FLS (Fig 1C). Rat FLS gave a 4-fold higher expression. Next, the bioactivity of hIFN-β produced by the ART-I02 vector was assessed in the FLS from the 5 different species. First, the biological effect hIFN-β on secretion of pro- and anti-inflammatory cytokines and matrix metalloproteinase (MMP)-3 in RA FLS supernatants was analyzed. In human RA FLS, TNF-α induced IL-8 and MMP-3 levels were significantly reduced in the presence of hIFN-β (80% and 60% respectively, P<0.05). Interleukin-6 levels were reduced by 60% only after stimulation with both TNF-α and IL-1β (P<0.01). Levels of anti-inflammatory cytokine IL-1 ra were increased 90% in the presence of hIFN-β (p<0.001) after TNF-α or TNF-α and IL-1β stimulation (Fig 2A). The primary cells used in this experiment have similar rates of response, but the different cell lines, especially FLS obtained from different RA patients, can differ significantly in absolute baseline levels and absolute levels of response. In order to be able to compare cytokine levels between cell lines, the levels of responses are presented in arbitrary units (AU, percentages). The actual levels (pg/ml) of each FLS cell line used are presented in the supplementary material (S1 and S2 Datasets).

To further assess hIFN-β bioactivity a quantitative gene reporter bioassay was used. Human type 1 IFN sensitive cells respond to the presence bio-active hIFN-β by producing luciferase. Human IFN-β bioactivity assessed through the quantitative gene reporter bioassay showed that supernatant of transduced activated RA FLS showed a clear positive effect on bioluminescence intensity of type 1 IFN sensitive cells (Fig 2B).

Since it is known that the bioactivity of human IFN-β is highly species-specific [4], we also investigated the effect on cytokines in FLS from different species. NHP FLS expressing hIFN-β derived from ART-I02 gene expression, showed a 35% decrease in IL-8 secretion (P<0.05) after stimulation with TNF-α (Fig 2C). The effect on IL-6 production showed a trend towards a reduction. Bioactivity of hIFN-β produced by NHP FLS measured by quantitative gene reporter bioassay gave similar results as obtained from RA FLS (Fig 2B). No biological effect of hIFN-β on cytokine production was observed in rabbit and rodent FLS as expected since hIFN-β is highly species specific.

#### Methotrexate influence on vector expression and bioactivity

**Fig 3:**
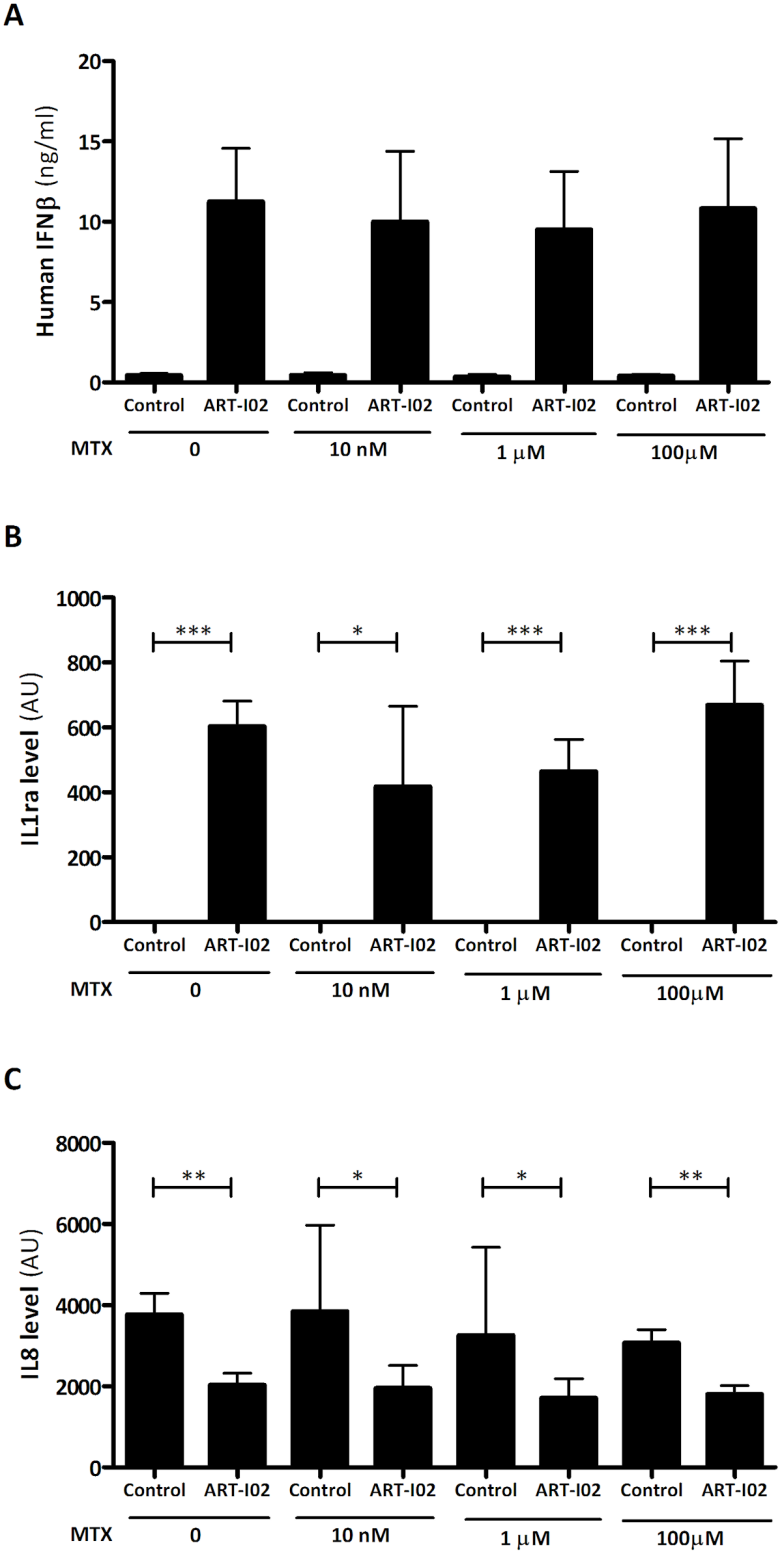
Effect of MTX on transgene expression and bioactivity in RA FLS. In the presence of MTX hIFN-β transgene expression (A) and bioactivity shown by change in IL-1ra (B) and IL-8 (C) remained unaltered in RA FLS. All samples were stimulated with TNF-α. Control cells were stimulated but not transduced with ART-I02. Data shown are mean + SEM (hIFN-β) and mean + SD (cytokines), N = 3–6 experiments, * indicates P<0.05, ** P<0.01 and *** P<0.001.

The effect of MTX on transduction efficiency and transgene bioactivity was investigated in RA FLS. Methotrexate is a frequently used disease modifying anti-rheumatic drug (DMARD). The influence of MTX on transduction efficiency is of importance, since many trials investigating new anti-rheumatic treatments include RA patients on MTX treatment. Previously, it was shown that AAV transduction efficiency in primary human fibroblasts was unaltered after 20 hour MTX pre-incubation (0.1 mM to 1 nM) [25]. However, the effect of continuous presence of MTX on AAV transduction in FLS has not been investigated. Methotrexate concentrations were chosen to mimic concentrations present in serum of RA patients on weekly MTX treatment. Orally administered MTX is known to result in widely inter-individual variable plasma concentrations. Peak values ranging between 0.3–0.8 μM are observed in a weekly dosing regimen, while plasma values decrease to <0.05 μM 24 hours after a single dose [26]. Synovial membrane concentrations are estimated to be about 10 fold higher than plasma concentrations [27]. Levels of transgene expression in the culture medium were unaffected in the presence of MTX in concentrations ranging from 10 nM to 100 uM (Fig 3A). Bioactivity of hIFN-β as determined by the effect on cytokine production, was also unaltered (Fig 3B and 3C).

### Vector Biodistribution in Lewis Rats

**Fig 4:**
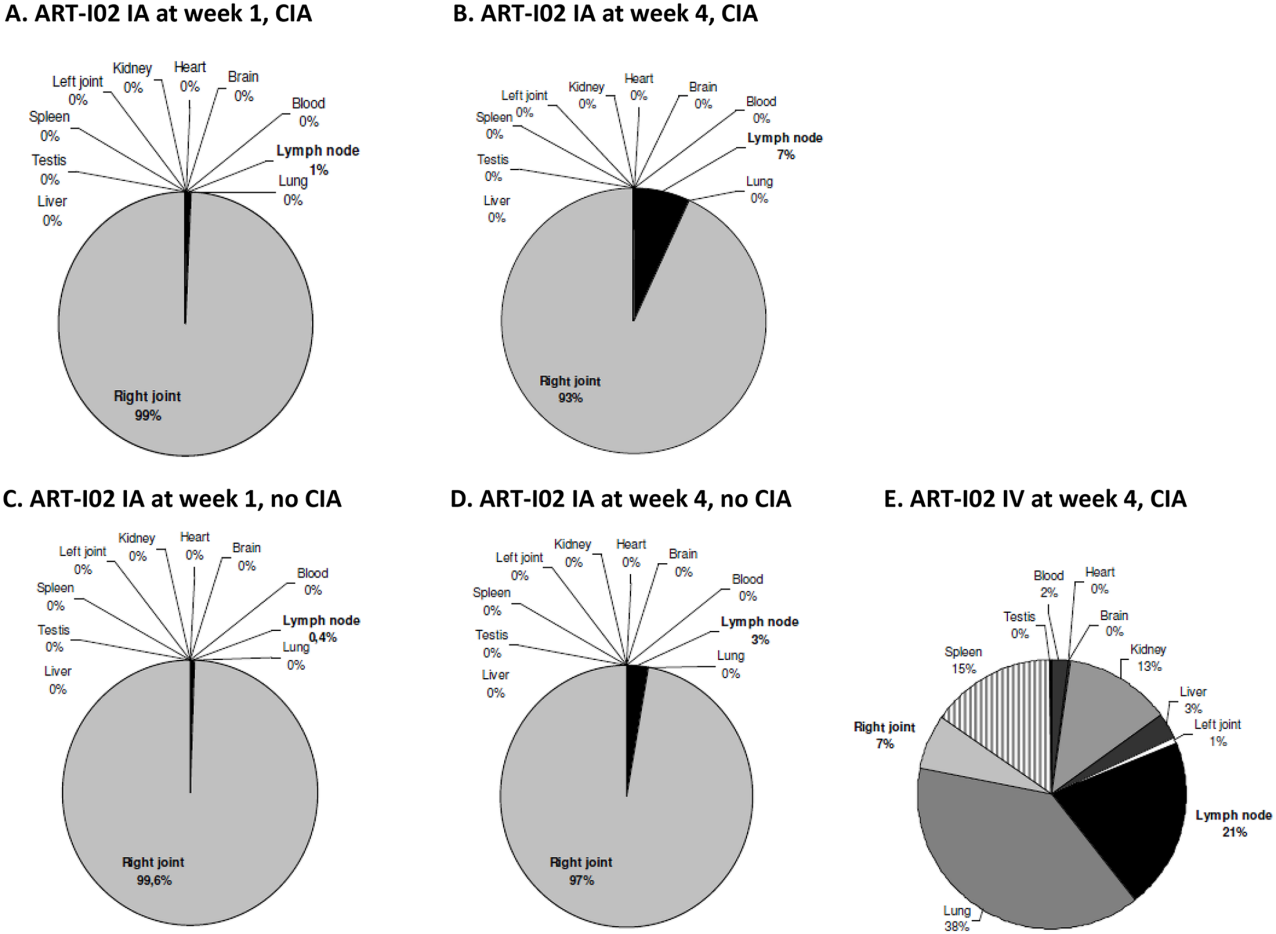
Vector biodistribution of ART-I02 in arthritic animals. Vectors were injected intra-articularly (IA) on day 14 after arthritis induction and vector DNA biodistribution was determined 1 and 4 weeks after administration by RT-PCR of a number of tissues (injected joint (right), non-injected joint (left), draining lymphnode, liver, lung, heart, testis, kidney, brain, spleen and blood) (N = 6 per group). Minimal vector spreading outside the joints was detected 1 and 4 weeks after intra-articular administration in animals with (A and B respectively) and without arthritis (C and D respectively). A different pattern was observed after intravenous (IV) administration of the vector, with almost 40% detected in the lungs (E). Data are shown as percentages of the total amount of vector retrieved. CIA, collagen induced arthritis.

**Fig 5:**
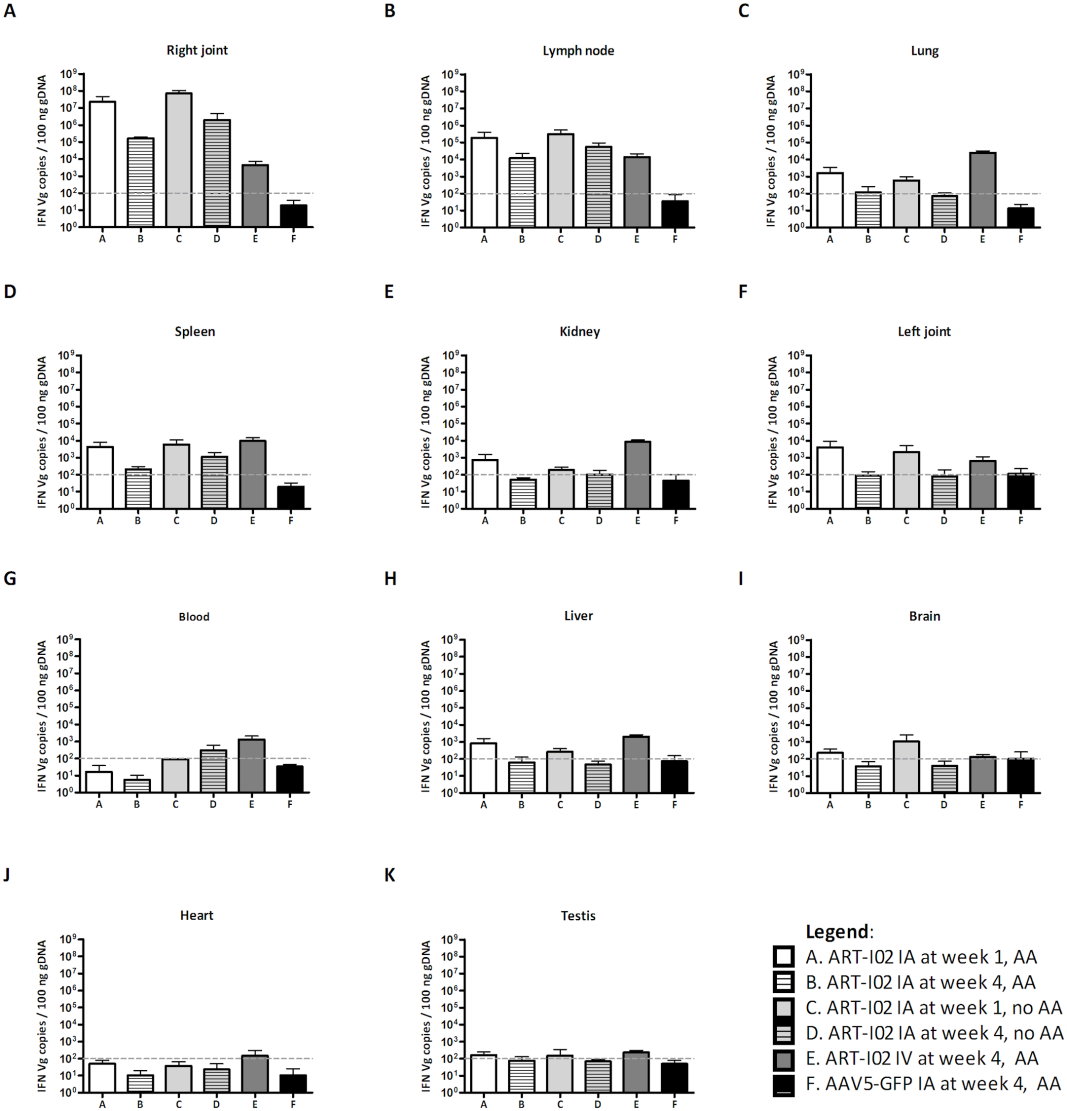
Vector copies of ART-I02 detected per tissue after intra-articular or intravenous administration. Besides in the intra-articularly injected right joint, high copy numbers were observed in draining lymph nodes and lung tissue. Intermediate numbers of vector copies were detected in spleen, kidney and blood, low numbers in liver, blood and brain. Levels in heart and testis were very low or below the detection limit of the assay. Data shown are mean + SD, graphs are shown with logarithmic scale. The 100 vg copies detection limit is depicted with a gray dashed line. N = 6 per group. GFP, green fluorescent protein; IA, intra-articular; IV, intravenous; week 1 or 4, number of weeks after vector administration; AA, adjuvant arthritis model.

For the biodistribution study rats were chosen since this species will also be used in the formal GLP toxicity and biodistribution studies. Biodistribution of ART-I02 after intra-articular injection was investigated in healthy and arthritic male rats since only male rats are susceptible for adjuvant-induced arthritis [28]. Biodistribution of viral genomes after administration of the vector (total 6X10^11^ vg/20 ul) into the right ankle joint at the onset of disease was compared to systemic (intravenous) administration of the same vector dose, both 1 and 4 weeks after injection. In the arthritic rats, the highest copy numbers of vector DNA were observed in the injected joints and in the surrounding draining lymph nodes. After 1 week, over 99% of vector DNA was detected in the injected joint (Figs 4A and 5A). Four weeks after injection of the vector, spreading of vector DNA to the draining lymph node of the injected joint was observed (6.8% +/- 6.1, Figs 4B and 5C). Spread to other organs after intra-articular administration was mainly seen in the spleen as well as the contralateral joint, with levels of vector genome copies around 2000–6000 vg copies/ 100 ng gDNA one week after vector administration and decreasing thereafter (Fig 5D and 5F). The virus can disseminate to the contralateral joint by trafficking of leukocytes or fibroblasts [29–31]. Other possible mechanisms could involve trafficking of dendritic cells or exosomes secreted by these cells which can contain vector DNA [32]. The communication of the dendritic cells between the two joints might be mediated by the draining lymph nodes of the involved joints.

For the other tissues, vector DNA copies were very low, with levels below 1000 vg copies / 100 ng DNA, one week after vector administration and undetectable levels at 4 weeks (Fig 5C, 5E and 5G–5K). Less than 0.01% of all vector genomes was detected in the gonads after 1 week and levels were below the detection limit 4 weeks after intra-articular administration (Fig 5K). Similar biodistribution was observed in non-arthritic compared to arthritic rats, where most of the vector DNA was detected in the injected joint one and 4 weeks after injection (99.6% and 97.3%, respectively) (see Fig 4C and 4D).

Since injection of vector directly into the target organ can lead to leakage of small quantities of vector into the blood circulation we evaluated the biodistribution after intravenous injection with an identical dose of the virus as the intra-articularly injected rats (Fig 4E). With maximum exposure, ART-I02 vector DNA could be detected mainly in the lung (38.5%), lymph nodes (20.7%), spleen (14.9%) and kidney (12.8%) and to a lower extent in the liver (3.1%) (Fig 5B–5E and 5H). Low levels of vector DNA were found in the gonads after systemic administration of the vector (0.3%).

### Transgene Expression in DBA-1 Mice

**Fig 6:**
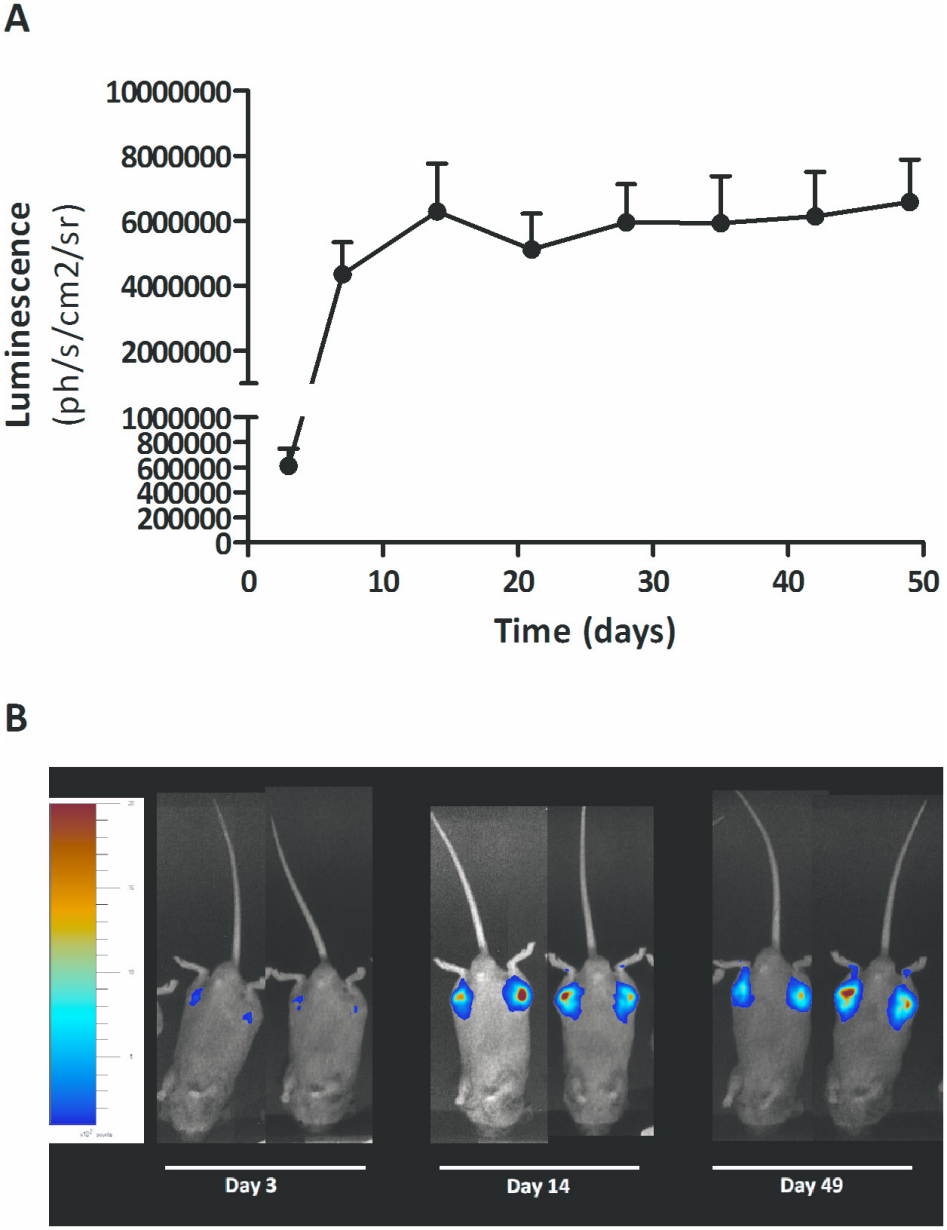
Luminescence of rAAV5-CMV-Fluc detected during longitudinal follow up after intra-articular administration. Expression was monitored for a total of 7 weeks. Expression is visible as early as the first imaging moment (day 3) and remained stable up till day 49 (A). Images over time of 2 representative animals are shown for day 3, 14 and 49 (end of experiment)(B). Data are shown as mean + SEM, N = 5.

In order to evaluate localized protein expression levels after intra-articular injection of the vector, an AAV5 vector expressing the *firefly luciferase* gene under control of a CMV promoter was generated (AAV5.CMV.FLuc) to allow for direct imaging of transgene expression *in vivo* over time. For the imaging experiments mice were chosen since rats could not be imaged in the In Vivo Imaging System (IVIS). In this rodent imaging study we observed sustained expression up until 7 weeks (Fig 6A), after which the experiment was terminated. Local intra-articular administration resulted in rapidly detectable transgene expression, already 3 days after vector administration. This expression was increasing up to 2 weeks after vector injection and then stable until the end of the experiment. We observed a slight non-significant decrease in expression three weeks after vector administration. Bioluminescence related to vector expression after intra-articular injection remained confined to the knee joint region (Fig 6B). Animals did not show luciferase expression anywhere in the abdomen or thorax. This phenomenon of initial high expression followed by a slight decline and subsequent stabilization of expression has been observed before in rodent models of arthritis [8,9]. The initial transduction of short-lived cells in an arthritic joint may contribute to this phenomenon [33]. Longer term expressing is most likely due to transduction of synovial tissue, including synovial fibroblasts and to a lesser degree +chondrocytes [8,12,34].

## Conclusions

Despite the extensive expansion in treatment options for RA patients during the last decade, complete remission is still achieved in only a minority of the patients, leaving most with at least monoarticular or oligoarticular disease activity [35]. In addition, there is still an unmet need for optimal local, intra-articular treatment in patients with monoarthritis or oligoarthritis who do not necessarily need systemic biological therapy and are not considered candidates for these expensive therapies (>15,000 euro/patient/year). For these patients local gene therapy can provide a patient-friendly treatment regimen resulting in more prolonged expression of therapeutic proteins after a single injection. The immunomodulatory protein IFN-β has been shown to hold great potential as a novel therapeutic and local approach.

In the *in vitro* potency studies it was clearly shown that FLS from mice, rats, rabbits, NHPs and RA patients could efficiently be transduced using rAAV5 as vector. In rabbits and rodents efficient expression but no bioactivity of hIFN-β was observed, as expected due to the high species-specificity of hIFN-β [4]. However, ART-I02 showed a clear anti-inflammatory effect in both RA and NHP FLS, supporting the use of collagen-induced arthritis in NHP as a model for the efficacy and non-clinical pharmacology-toxicity evaluation of gene therapy for RA. Since rodent FLS can be transduced by rAAV5, these species can also be used in formal GLP biodistribution and toxicity studies of ART-I02 as required before the first injection in humans. The presence of MTX did not alter the expression levels of hIFN-β in *in vitro* transduction experiments. The finding that MTX does not influence transduction efficiency or transgene expression is important since most of the RA patients included in the clinical trial will be under MTX therapy, as this is standard in evaluating new treatments for RA.

Gene therapy investigators have been generally concerned about the diffusion of the vector to the major organs. The rat biodistribution study demonstrated a clear local biodistribution pattern after intra-articular administration of ART-I02, compared to systemic administration. Minimal biodistribution could be observed one week after intra-articular injection and stable long-term expression was demonstrated in the joint. Most spreading occurred to draining lymph nodes in the arthritic rats with minimal spreading to other organs, including the brain and testes. The biodistribution study showed a similar pattern in arthritic and non-arthritic rats. This is important since the latter will be used in formal GLP studies. The studies presented in this paper reveal that the diffusion of ART-I02 vector to major organs is not seen after local administration of rAAV5 vector into the inflamed knee joint, contributing to the safety of local gene therapy as a new treatment option for RA.

We demonstrated that local intra-articular administration of a rAAV5 vector resulted in rapidly detectable transgene expression, already 3 days after vector administration. Previous imaging studies have reported a slower onset of expression of rAAV5-CMV-Fluc at a dose of 1x10^11^ vg after tail vein administration, with no expression detected on day 7 and the first expression detectable after 14 days [36]. Local administration into the intended tissue might improve tissue transduction of the vector and be the reason for earlier onset of transgene expression, compared to systemic non-targeted administration. Also, a similar dose (1.71x10^11^ vg) administered locally into a smaller area may have contributed to the level of expression.

Previous studies on hemophilia B in larger animal models have confirmed the potential of rAAV vectors to achieve long-term expression. AAV2-mediated liver-directed gene therapy corrected the hemophilia phenotype in dogs for more than 8 years [37]. Also, in nonhuman primates AAV5 and AAV8 factor IX expression remained stable over a period of more than 5 years [38]. Although our duration of follow up was relatively short, the expression pattern appears stable and not declining over time. These data suggest that local inflammation does not negatively influence transgene expression. Currently we are performing longer term follow-up studies in arthritic animals up to 6 months.

In conclusion, we have demonstrated that our vector, ART-I02, can produce bioactive hIFNβ in RA FLS and can modulate inflammation. In addition, we have shown that ART-I02 can efficiently transduce FLS from other species. Our *in vivo* experiments support the use of intra-articular delivery of rAAV5 as an efficient system for expression of a therapeutic protein in the joint, with only limited biodistribution of the vector to peripheral organs. Together with efficacy data in rodents using a species specific IFNβ construct [6,12] and the fact that a limited number of RA patients express neutralizing antibodies to AAV5 [11], our findings support the progress towards a phase I clinical trial for the local treatment of arthritis in patients with RA.

## Supporting Information

S1 Dataset Raw data of Figs 1 and 2. (XLS)

S2 Dataset Raw data of Fig 3. (XLS)

S3 Dataset Raw data of Figs 4, 5 and S1 Fig. (XLS)

S4 Dataset Raw data of Fig 6. (XLS)

S1 Fig Vector copies of ART-I02 detected per tissue after intra-articular or intravenous administration. To provide additional insight into the data of Fig 5, in this figure graphs are shown with adjusted y-axes. Graphs A-C are shown with logarithmic scale. Graphs D-K are shown on a linear scale, with different maximum values per row. Data shown are mean + SD. The 100 vg copies detection limit is depicted with a gray dashed line.
(TIF)

S1 Table Overview of transduction experiments. (DOC)

S2 Table Group set up of the biodistribution study in rats. Vg, viral genomes.
(DOC)

S3 Table Group set up of the imaging study in mice. Vg, viral genomes.
(DOC)

## References

[1] Tak PP. IFN-beta in rheumatoid arthritis. Front Biosci. 2004; 9: 3242–3247. 15353352 10.2741/1475

[2] Van Holten J, Reedquist K, Sattonet-Roche P, Smeets TJ, Plater-Zyberk C, Vervoordeldonk MJ, et al Treatment with recombinant interferon-beta reduces inflammation and slows cartilage destruction in the collagen-induced arthritis model of rheumatoid arthritis. Arthritis Res Ther. 2004;6: R239–R249. 15142270 10.1186/ar1165 PMC416442

[3] Triantaphyllopoulos KA, Williams RO, Tailor H, Chernajovsky Y. Amelioration of collagen-induced arthritis and suppression of interferon-gamma, interleukin-12, and tumor necrosis factor alpha production by interferon-beta gene therapy. Arthritis Rheum. 1999;42: 90–99. 9920019 10.1002/1529-0131(199901)42:1<90::AID-ANR12>3.0.CO;2-A

[4] Tak PP, Hart BA, Kraan MC, Jonker M, Smeets TJ, Breedveld FC. The effects of interferon beta treatment on arthritis. Rheumatology (Oxford). 1999;38: 362–369. 10378715 10.1093/rheumatology/38.4.362

[5] Van Holten J, Pavelka K, Vencovsky J, Stahl H, Rozman B, Genovese M, et al A multicentre, randomised, double blind, placebo controlled phase II study of subcutaneous interferon beta-1a in the treatment of patients with active rheumatoid arthritis. Ann Rheum Dis. 2005;64: 64–69. 15242865 10.1136/ard.2003.020347 PMC1755211

[6] Adriaansen J, Kuhlman RR, van Holten J, Kaynor C, Vervoordeldonk MJ, Tak PP. Intraarticular interferon-beta gene therapy ameliorates adjuvant arthritis in rats. Hum Gene Ther. 2006;17: 985–996. 16984225 10.1089/hum.2006.17.985

[7] Russell DW, Alexander IE, Miller AD. DNA synthesis and topoisomerase inhibitors increase transduction by adeno-associated virus vectors. Proc Natl Acad Sci U S A. 1995;92: 5719–5723. 7777575 10.1073/pnas.92.12.5719 PMC41768

[8] Adriaansen J, Tas SW, Klarenbeek PL, Bakker AC, Apparailly F, Firestein GS, et al Enhanced gene transfer to arthritic joints using adeno-associated virus type 5: implications for intra-articular gene therapy. Ann Rheum Dis. 2005;64: 1677–1684. 15878906 10.1136/ard.2004.035063 PMC1755308

[9] Apparailly F, Khoury M, Vervoordeldonk MJ, Adriaansen J, Gicquel E, Perez N, et al Adeno-associated virus pseudotype 5 vector improves gene transfer in arthritic joints. Hum Gene Ther. 2005;16: 426–434. 15871674 10.1089/hum.2005.16.426

[10] Boutin S, Monteilhet V, Veron P, Leborgne C, Benveniste O, Montus MF, et al Prevalence of serum IgG and neutralizing factors against adeno-associated virus (AAV) types 1, 2, 5, 6, 8, and 9 in the healthy population: implications for gene therapy using AAV vectors. Hum Gene Ther. 2010;21: 704–712. 10.1089/hum.2009.182 20095819

[11] Mingozzi F, Chen Y, Edmonson SC, Zhou S, Thurlings RM, Tak PP, et al Prevalence and pharmacological modulation of humoral immunity to AAV vectors in gene transfer to synovial tissue. Gene Ther. 2013;20:417–424. 10.1038/gt.2012.55 22786533 PMC3473155

[12] Adriaansen J, Fallaux FJ, de Cortie CJ, Vervoordeldonk MJ, Tak PP. Local delivery of beta interferon using an adeno-associated virus type 5 effectively inhibits adjuvant arthritis in rats. J Gen Virol. 2007;88: 1717–1721. 17485531 10.1099/vir.0.82603-0 PMC2884954

[13] Nathwani AC, Gray JT, McIntosh J, Ng CY, Zhou J, Spence Y, et al Safe and efficient transduction of the liver after peripheral vein infusion of self-complementary AAV vector results in stable therapeutic expression of human FIX in nonhuman primates. Blood. 2007;109: 1414–1421. 17090654 10.1182/blood-2006-03-010181 PMC1794053

[14] Paneda A, Vanrell L, Mauleon I, Crettaz JS, Berraondo P, Timmermans EJ, et al Effect of adeno-associated virus serotype and genomic structure on liver transduction and biodistribution in mice of both genders. Hum Gene Ther. 2009;20: 908–917. 10.1089/hum.2009.031 19419275

[15] Favaro P, Finn JD, Siner JI, Wright JF, High KA, Arruda VR. Safety of liver gene transfer following peripheral intravascular delivery of adeno-associated virus (AAV)-5 and AAV-6 in a large animal model. Hum Gene Ther. 2011;22: 843–852. 10.1089/hum.2010.155 21126217 PMC3135234

[16] Matsushita T, Elliger S, Elliger C, Podsakoff G, Villarreal L, Kurtzman GJ, et al Adeno-associated virus vectors can be efficiently produced without helper virus. Gene Ther. 1998; 5: 938–945. 9813665 10.1038/sj.gt.3300680

[17] Gao GP, Alvira MR, Wang L, Calcedo R, Johnston J, Wilson JM. Novel adeno-associated viruses from rhesus monkeys as vectors for human gene therapy. Proc Natl Acad Sci U S A. 2002;99: 11854–11859. 12192090 10.1073/pnas.182412299 PMC129358

[18] van de Sande MG, Gerlag DM, Lodde BM, van Baarsen LG, Alivernini S, Codullo V, et al Evaluating antirheumatic treatments using synovial biopsy: a recommendation for standardisation to be used in clinical trials. Ann Rheum Dis. 2011;70: 423–427. 10.1136/ard.2010.139550 21109518

[19] Tak PP, Gerlag DM, Aupperle KR, van de Geest DA, Overbeek M, Bennett BL, et al Inhibitor of nuclear factor kappaB kinase beta is a key regulator of synovial inflammation. Arthritis Rheum. 2001;44: 1897–1907. 11508443 10.1002/1529-0131(200108)44:8<1897::AID-ART328>3.0.CO;2-4

[20] Ruijter JM, Thygesen HH, Schoneveld OJ, Das AT, Berkhout B, Lamers WH. Factor correction as a tool to eliminate between-session variation in replicate experiments: application to molecular biology and retrovirology. Retrovirology. 2006;3: 2 1742–1746. 16398936 10.1186/1742-4690-3-2 PMC1368993

[21] Gao G, Vandenberghe LH, Wilson JM. New recombinant serotypes of AAV vectors. Curr Gene Ther. 2005;5: 285–297. 15975006 10.2174/1566523054065057

[22] Yan Z, Zak R, Zhang Y, Ding W, Godwin S, Munson K, et al Distinct classes of proteasome-modulating agents cooperatively augment recombinant adeno-associated virus type 2 and type 5-mediated transduction from the apical surfaces of human airway epithelia. J Virol. 2004;78: 2863–2874. 14990705 10.1128/JVI.78.6.2863-2874.2004 PMC353734

[23] Yan Z, Lei-Butters DC, Zhang Y, Zak R, Engelhardt JF. Hybrid adeno-associated virus bearing nonhomologous inverted terminal repeats enhances dual-vector reconstruction of minigenes in vivo. Hum Gene Ther. 2007;18: 81–87. 17181493 10.1089/hum.2006.128 PMC2121583

[24] Zhang T, Hu J, Ding W, Wang X. Doxorubicin augments rAAV-2 transduction in rat neuronal cells. Neurochem Int. 2009;55: 521–528. 10.1016/j.neuint.2009.05.005 19450628

[25] Alexander IE, Russell DW, Miller AD. DNA-damaging agents greatly increase the transduction of nondividing cells by adeno-associated virus vectors. J Virol. 1994;68: 8282–8287. 7966621 10.1128/jvi.68.12.8282-8287.1994 PMC237296

[26] Kremer JM. Toward a better understanding of methotrexate. Arthritis Rheum. 2004;50: 1370–1382. 15146406 10.1002/art.20278

[27] Bologna C, Edno L, Anaya JM, Canovas F, Vanden Berghe M, Jorgensen C, et al Methotrexate concentrations in synovial membrane and trabecular and cortical bone in rheumatoid arthritis patients. Arthritis Rheum. 1994;37: 1770–1773. 7986223 10.1002/art.1780371210

[28] Bevaart L, Vervoordeldonk MJ, Tak PP. Evaluation of therapeutic targets in animal models of arthritis: how does it relate to rheumatoid arthritis? Arthritis Rheum. 2010;62: 2192–2205. 10.1002/art.27503 20506322

[29] Lechman ER, Jaffurs D, Ghivizzani SC, Gambotto A, Kovesdi I, Mi Z, et al Direct adenoviral gene transfer of viral IL-10 to rabbit knees with experimental arthritis ameliorates disease in both injected and contralateral control knees. J Immunol. 1999; 163: 2202–2208. 10438962

[30] Lechman ER, Keravala A, Nash J, Kim SH, Mi Z, Robbins PD. The contralateral effect conferred by intra-articular adenovirus-mediated gene transfer of viral IL-10 is specific to the immunizing antigen. Gene Ther. 2003;10: 2029–2035. 14566362 10.1038/sj.gt.3302109

[31] Lefevre S, Knedla A, Tennie C, Kampmann A, Wunrau C, Dinser R, et al Synovial fibroblasts spread rheumatoid arthritis to unaffected joints. Nat Med. 2009;15: 1414–1420. 10.1038/nm.2050 19898488 PMC3678354

[32] Evans CH, Robbins PD, Ghivizzani SC, Wasko MC, Tomaino MM, Kang R, et al Gene transfer to human joints: progress toward a gene therapy of arthritis. Proc Natl Acad Sci U S A. 2005;102: 8698–8703. 15939878 10.1073/pnas.0502854102 PMC1150836

[33] Gouze E, Gouze JN, Palmer GD, Pilapil C, Evans CH, Ghivizzani SC. Transgene persistence and cell turnover in the diarthrodial joint: implications for gene therapy of chronic joint diseases. Mol Ther. 2007;15: 1114–1120. 17440444 10.1038/sj.mt.6300151

[34] Watson RS, Broome TA, Levings PP, Rice BL, Kay JD, Smith AD, et al scAAV-mediated gene transfer of interleukin-1-receptor antagonist to synovium and articular cartilage in large mammalian joints. Gene Ther. 2013;20: 670–677. 10.1038/gt.2012.81 23151520 PMC3577988

[35] Tak PP, Kalden JR. Advances in rheumatology: new targeted therapeutics. Arthritis Res Ther. 2011;13 Suppl 1: S5 1478-6354-13-S1-S5. 10.1186/1478-6354-13-S1-S5 21624184 PMC3123966

[36] Zincarelli C, Soltys S, Rengo G, Rabinowitz JE. Analysis of AAV serotypes 1–9 mediated gene expression and tropism in mice after systemic injection. Mol Ther. 2008;16: 1073–1080. 10.1038/mt.2008.76 18414476

[37] Niemeyer GP, Herzog RW, Mount J, Arruda VR, Tillson DM, Hathcock J, et al Long-term correction of inhibitor-prone hemophilia B dogs treated with liver-directed AAV2-mediated factor IX gene therapy. Blood. 2009;113: 797–806. 10.1182/blood-2008-10-181479 18957684 PMC2630266

[38] Nathwani AC, Rosales C, McIntosh J, Rastegarlari G, Nathwani D, Raj D, et al Long-term safety and efficacy following systemic administration of a self-complementary AAV vector encoding human FIX pseudotyped with serotype 5 and 8 capsid proteins. Mol Ther. 2011;19: 876–885. 10.1038/mt.2010.274 21245849 PMC3098629

