# Supplementary figures and images for: Preclinical Potency and Biodistribution Studies of an AAV 5 Vector Expressing Human Interferon-β (ART-I02) for Local Treatment of Patients with Rheumatoid Arthritis
Source: PLoS One. 2015 Jun 24;10(6):e0130612. doi: 10.1371/journal.pone.0130612 (PMC4479517; doi:10.1371/journal.pone.0130612)

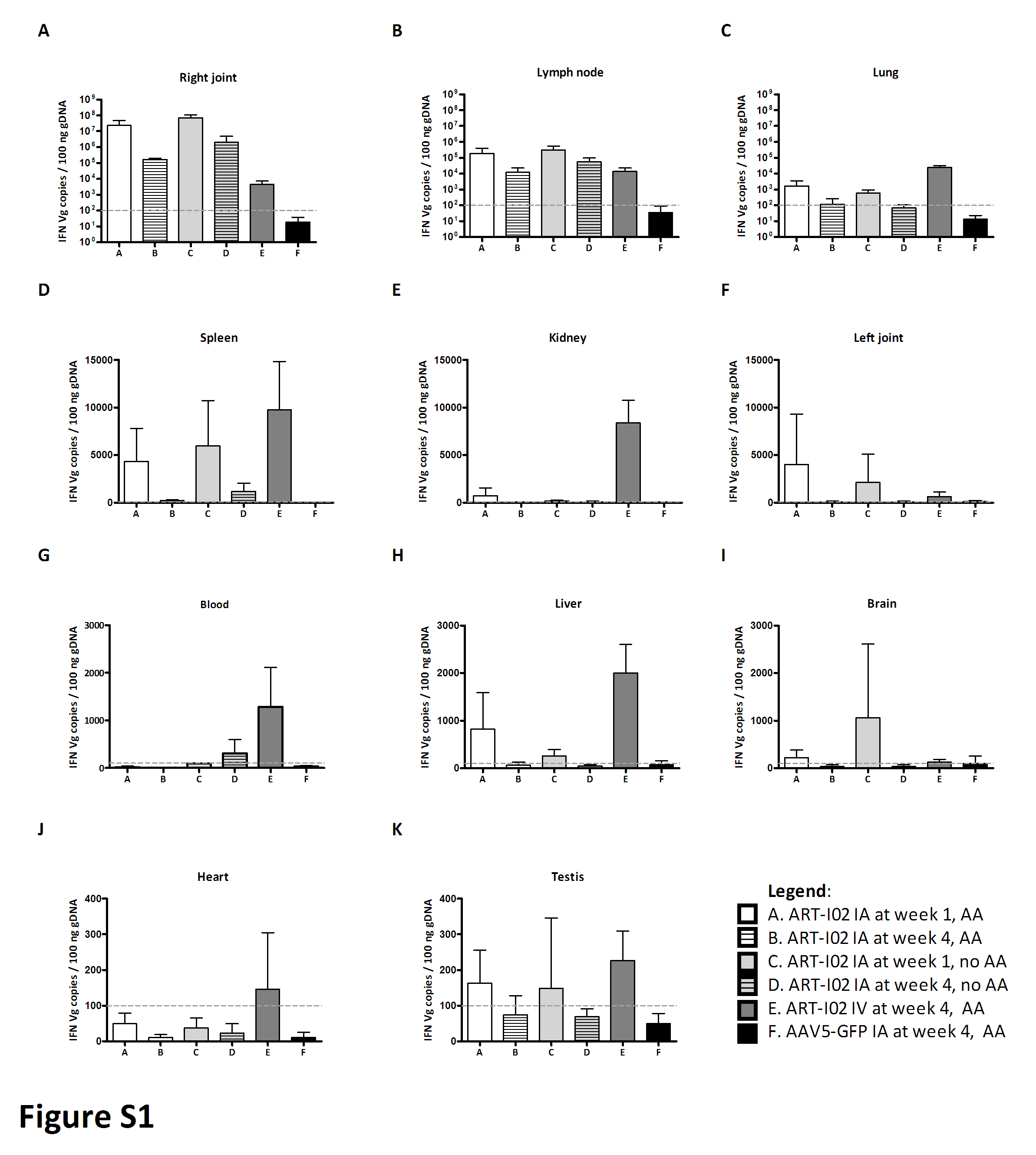

Supplement: S1 Fig — To provide additional insight into the data of Fig 5, in this figure graphs are shown with adjusted y-axes. Graphs A-C are shown with logarithmic scale. Graphs D-K are shown on a linear scale, with different maximum values per row. Data shown are mean + SD. The 100 vg copies detection limit is depicted with a gray dashed line. (TIF) [file pone.0130612.s005.tif]
