# Supplementary material for: Preclinical Potency and Biodistribution Studies of an AAV 5 Vector Expressing Human Interferon-β (ART-I02) for Local Treatment of Patients with Rheumatoid Arthritis
Source: PLoS One. 2015 Jun 24;10(6):e0130612. doi: 10.1371/journal.pone.0130612 (PMC4479517; doi:10.1371/journal.pone.0130612)
Supplement: S1 Table — (DOC) [file pone.0130612.s006.doc]

**S1 Table**

| **Figure** | **Cells** | **Vector** | **Stimulation** | **Incubation** | **Outcome** | **Method** | **Other** |  |
| --- | --- | --- | --- | --- | --- | --- | --- | --- |
| 1A | RA FLS | ART-I02 | TNFα | 48 hours | hIFN-β level | ELISA | +/- doxorubicin | |
| 1B | RA FLS | ART-I02 | TNFα and/or IL-1β | 48 hours | hIFN-β level | ELISA | - |  |
| 1C | RA, NHP, mouse,  rabbit, rat FLS | ART-I02 | TNFα | 48 hours | hIFN-β level | ELISA | - |  |
| 2A | RA FLS | ART-I02 | TNFα (+IL-1β) | 48 hours | Cytokine production | ELISA | - |  |
| 2B | RA and NHP FLS | ART-I02 | TNFα | 48 hours | hIFN-β bioactivity | Quantitative gene reporter bioassay | | |
| 2C | NHP FLS | ART-I02 | TNFα | 48 hours | Cytokine production | ELISA | - |  |
| 3A | RA FLS | ART-I02 | TNFα | 48 hours | hIFN-β level | | ELISA | | --- | | +/- Methotrexate | |
| 3B-C | RA FLS | ART-I02 | TNFα | 48 hours | Cytokine production | ELISA | (10 nM, 1 µM, 100 µM) | |
