# Supplementary material for: Preclinical Potency and Biodistribution Studies of an AAV 5 Vector Expressing Human Interferon-β (ART-I02) for Local Treatment of Patients with Rheumatoid Arthritis
Source: PLoS One. 2015 Jun 24;10(6):e0130612. doi: 10.1371/journal.pone.0130612 (PMC4479517; doi:10.1371/journal.pone.0130612)
Supplement: S2 Table — Vg, viral genomes. (DOC) [file pone.0130612.s007.doc]

**S2 Table**

| **Group** | **Name** | **Nr of animals** | **Vector** | **Dose** | **Route of administration** | **Follow up** | **Arthritis** | **Outcome** |
| --- | --- | --- | --- | --- | --- | --- | --- | --- |
| 1 | IFN-ia-1-art | 6 | ART-I02 | 6x10e11 vg in 20 ul | Intra-articular | 1 week | | Yes | | --- | |  |
| 2 | IFN-ia-4-art | 6 | ART-I02 | 6x10e11 vg in 20 ul | Intra-articular | 4 weeks | Yes | Biodistribution |
| 3 | IFN-ia-1-no art | 6 | ART-I02 | 6x10e11 vg in 20 ul | Intra-articular | 1 weeks | No | by RT-PCR for |
| 4 | IFN-ia-4-no art | 6 | ART-I02 | 6x10e11 vg in 20 ul | Intra-articular | 4 weeks | No | vector DNA |
| 5 | IFN-iv-4-art | 6 | ART-I02 | 6x10e11 vg in 200 ul | Intravenous | 4 weeks | Yes |  |
| 6 | GFP-ia-4-art | 6 | AAV5.CMV.GFP | 8x10e10 vg in 20 ul | Intra-articular | 4 weeks | Yes |  |
