# Supplementary material for: Preclinical Potency and Biodistribution Studies of an AAV 5 Vector Expressing Human Interferon-β (ART-I02) for Local Treatment of Patients with Rheumatoid Arthritis
Source: PLoS One. 2015 Jun 24;10(6):e0130612. doi: 10.1371/journal.pone.0130612 (PMC4479517; doi:10.1371/journal.pone.0130612)
Supplement: S3 Table — Vg, viral genomes. (DOC) [file pone.0130612.s008.doc]

**S3 Table**

| **Group** | **Name** | **Nr of animals** | **Vector** | **Dose** | **Route of administration** | **Follow up** | **Arthritis** | **Outcome** |
| --- | --- | --- | --- | --- | --- | --- | --- | --- |
| 1 | Fluc-ia-no-art | 5 | rAAV5.CMV.FLUC | 3.42x10e10 vg in 20 ul | Intra-articular | 7 weeks | Yes | Luminescence |
